# Electrically active hydrogels based on PEDOT:PSS for neural cultures

**DOI:** 10.1039/d5tc02708j

**Published:** 2025-10-28

**Authors:** Liwen Wang, Yannick Hajee, Jean-Philippe Frimat, Mani Diba, Achilleas Savva

**Affiliations:** a Department of Microelectronics, Faculty of Electrical Engineering, Computer Science and Mathematics, Delft University of Technology Delft the Netherlands a.savva@tudelft.nl; b Regenerative Biomaterials–Dentistry, Research Institute for Medical Innovation, Radboud University Medical Center Nijmegen the Netherlands; c Department of Human Genetics, Leiden University Medical Center Leiden the Netherlands

## Abstract

Electrically active hydrogels are attracting significant interest as biohybrid materials for electrical interfacing with biological tissues. Here, we report the development of electrically active hydrogels, specifically engineered for *in vitro* neural cell cultures. The hydrogels’ matrix comprises a viscoelastic alginate primary network, interpenetrated by a secondary network formed by the neural cell-adhesive protein, laminin. Conducting poly(3,4-ethylenedioxythiophene):polystyrene sulfonate (PEDOT:PSS) particles are embedded throughout the hydrogel matrix, serving as the electrically active filler phase. Oscillatory rheology confirmed the viscoelastic nature of the composite hydrogels, with storage and loss moduli in the range of 1–10 kPa, suitable for neural tissue interfacing. The hydrogels exhibited high optical transparency across the visible spectrum. At a wavelength of 500 nm, transmission exceeded 45% for 400 µm thick hydrogels and was further enhanced to over 60% by reducing the hydrogel thickness to 150 µm. We established a reproducible protocol for electrochemical impedance spectroscopy and cyclic voltammetry measurements, demonstrating that the incorporation of PEDOT:PSS significantly enhanced both conductivity and charge storage capacitance of hydrogel films. The alginate–laminin–PEDOT:PSS hydrogels demonstrated excellent operational stability, maintaining consistent electrochemical performance over 80 charging/discharging cycles and remaining structurally and functionally stable under cell culture conditions for over four weeks. Cortical neuron cultures derived from human induced pluripotent stem cells prove the stability and cytocompatibility of our proposed hydrogels for over 28 days in culture. Collectively, these results highlight the potential of electrically active hydrogels loaded with PEDOT:PSS as soft, bioelectronic interfaces for neural engineering applications.

## Abstract 2

Electrically active hydrogels are attracting significant interest as biohybrid materials for electrical interfacing with biological tissues.

## Graphical abstract

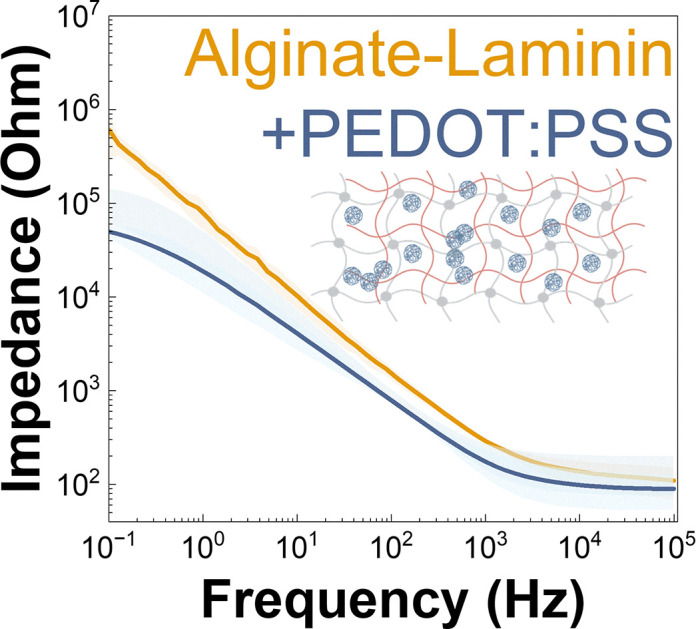

## Introduction

Bioelectronic materials and devices are emerging as promising platforms for monitoring and stimulating *in vitro* cell cultures.^1,2^ These platforms have advanced our understanding of complex biological processes associated with neuron electrophysiology guiding the development of future clinical treatments.^3^ Conventional bioelectronic *in vitro* models are based on 2D cell cultures, where a cell monolayer is formed and adhered to protein-coated microelectrode arrays.^4,5^ However, these systems fail to replicate the physical and chemical complexity of the human biology, and often result in cells with different functionality compared with cells that grow *in vivo*.^1^

3D human-like *in vitro* systems have been proven crucial for stem cell engineering and neuron regeneration.^6^ These systems have significantly benefited from the development of soft biomaterials, particularly hydrogels.^7,8^ Such biomaterials can replicate the mechanical properties of neural tissue (*i.e.* soft tissue with Young's moduli ∼1 kPa) and deliver biochemical cues that are crucial for neural growth in the body. Hydrogels made of extracellular matrix (ECM) components, or blends with other polymeric materials such as alginate, have been extensively used to develop biomimetic 3D neural cultures. These hydrogels provide a supportive environment that mimics the natural cellular surroundings, which is crucial for studying neural behavior and developing effective disease treatments.^9^ Alginate-based hydrogels can be fine-tuned by controlling the molecular weight of alginate chains as well as crosslinking density to produce hydrogels with adjustable viscoelasticity, rendering them excellent biomaterial candidates for *in vitro* 3D neural cultures.^10,11^

More recently, electrically active hydrogels are gaining attention due to their ability to replicate the bioelectrical patterns of neural tissue, while preserving excellent mechanical and chemical compatibility.^12^ These biohybrid electronic materials can be tailored to construct highly biomimetic electrodes for neural recordings,^13^ and injectable conducting hydrogels for spinal cord injuries.^14^ Electrically active hydrogels for 3D *in vitro* models were found to enhance the differentiation and maturation of stem cells towards neural lineage, due to the hydrogels’ inherent electrical properties.^15,16^ These results are highly promising and demonstrate how further development of electrically active hydrogels can be optimized to guide the differentiation and maturation of stem cells into neural tissue, opening new opportunities for bioelectronic applications in neural engineering. The most commonly used strategy to render hydrogel matrices electroconductive is by blending electroconductive particles with hydrogel forming polymers, such as alginate and gelatin.^17^ Conducting materials such as carbon nanotubes,^15^ graphene,^18^ and silver nanowires^19^ have been used to create conducting hydrogels that can support the development of 3D neural cultures, *in vitro*.

Electrically active hydrogels made with the conducting polymer PEDOT:PSS (*i.e.* poly(3,4-ethylenedioxythiophene) polystyrene sulfonate) are gaining attention.^20,21^ The chemistry of PEDOT:PSS and its stable water dispersions that are commercially available in large quantities provide flexibility in preparation and casting of high cytocompatibility hydrogels, with tunable properties. For example, Spencer *et al.* used PEDOT:PSS particles dispersed in a gelatin methacryloyl (GelMA) matrix to obtain an electrically active hydrogel system with cells encapsulated, proving its potential in 3D tissue guidance through electrical stimulation.^22^ Recently, Rutz *et al.* developed 3D printable PEDOT:PSS-based hydrogels with an ionic liquid as a cross-linker.^23^ The excellent cytocompatibility of PEDOT:PSS is already demonstrated in several studies that developed electronic scaffolds able to host 3D human stem cell^24^ and neural cultures.^25^

Here, we report the development of electrically active hydrogel systems made with a combination of PEDOT:PSS embedded in a viscoelastic matrix formed by sodium alginate and supported by a secondary network of the neural protein laminin. A comprehensive analysis of the hydrogels’ key properties is presented, confirming their excellent compatibility with the demands of sensitive stem cell–derived neural cultures. We showed that high optical transparency in the visible spectrum can be maintained even after the PEDOT:PSS particle inclusion in the hydrogel matrix, which can be further fine-tuned by controlling the thickness of the hydrogels in the range between 150 µm and 400 µm. Furthermore, we examined in detail the mechanical properties of the hydrogels with oscillatory rheology. The storage moduli (*G*′) of the hydrogels with PEDOT:PSS are shown to be in the order of 5 kPa, which verifies the compatibility of the hydrogels with the mechanical properties of neural tissue.^26^ Detailed electrochemical characterization reveals an increase in electrical conductivity and charge storage capacitance of the hydrogels containing PEDOT:PSS compared with pure-alginate/laminin hydrogels, and stable electrochemical operation for more than 80 charging cycles. Finally, the hydrogels are highly stable under stem cell culture conditions for more than 28 days, and highly biocompatible as proved by live/dead assays of cortical neurons derived from human induced pluripotent stem cell cultures.

## Results and discussion

**Fig. 1:**
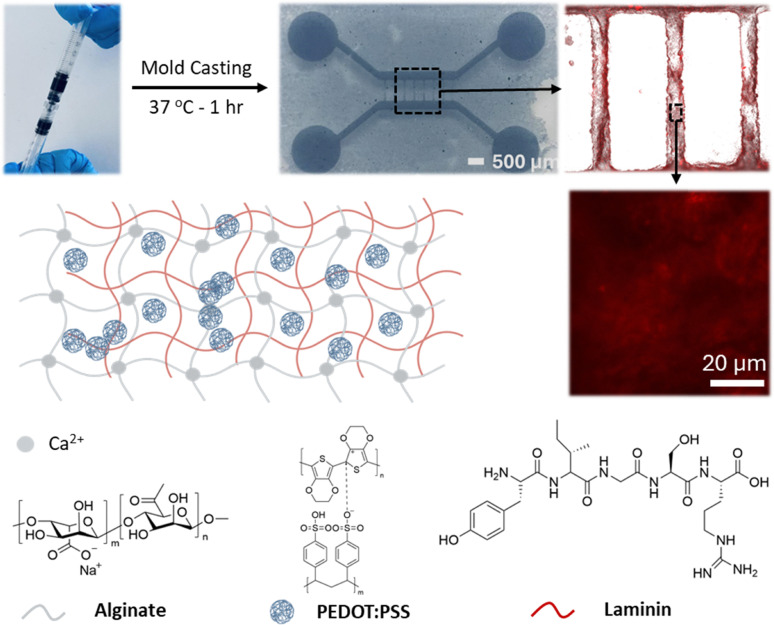
A schematic representation of the alginate–laminin-PEDOT:PSS hydrogel system with an interpenetrating network forming and PEDOT:PSS nanoparticles included in the matrix. The particles are partially percolated, forming electrically active pathways within the ionically conducting hydrogel matrix. The top row illustrates the preparation process, where two syringes are interconnected using a Luer-lock connector to mix the hydrogel precursors – the first one contains the sodium alginate and laminin and the water dispersion of PEDOT:PSS – and the second contains CaSO_4_ to initiate gelation upon casting at 37 °C in custom-made molds. The top row also includes a digital image of the patterned hydrogel and a fluorescence image showing laminin distribution.

The electrically active hydrogels were made by mixing the water dispersion of PEDOT:PSS with water solutions of alginate and laminin, and casting them in tailor-made molds with controlled thickness, as described in the Experimental section. A schematic of alginate–laminin-PEDOT:PSS hydrogel network is illustrated in Fig. 1. The sodium alginate forms the primary network of the hydrogel upon crosslinking with Ca^2+^ ions.^27,28^ To prove the formation of the laminin interpenetrating network we used a laminin tagged with a fluorescent label (*i.e.* red fluorescent, rhodamine – see the Experimental section) and imaged the electrically active hydrogels with fluorescence microscopy. As shown in Fig. 1, a uniform fluorescence distribution across a wide surface is observed, proving the stable formation of the laminin network within the hydrogel. The conducting PEDOT:PSS particles are expected to be entangled within this interpenetrating network formed by alginate and laminin, forming partially connected electrically conducting networks. Following this understanding, we fine-tuned the casting and gelation process of the hydrogels by using slowly dissolving CaSO_4_ particles to control the release rate of calcium ions.^27,29^ This approach prevents excessively fast gelation, and allows for the controllable casting of the hydrogels in custom-made 3D printed molds of different shapes and dimensions, as shown in Fig. 1. With the same approach, and by tuning the height of the molds, different hydrogel thicknesses can be achieved, spanning from 150 µm to 10 mm.

**Fig. 2:**
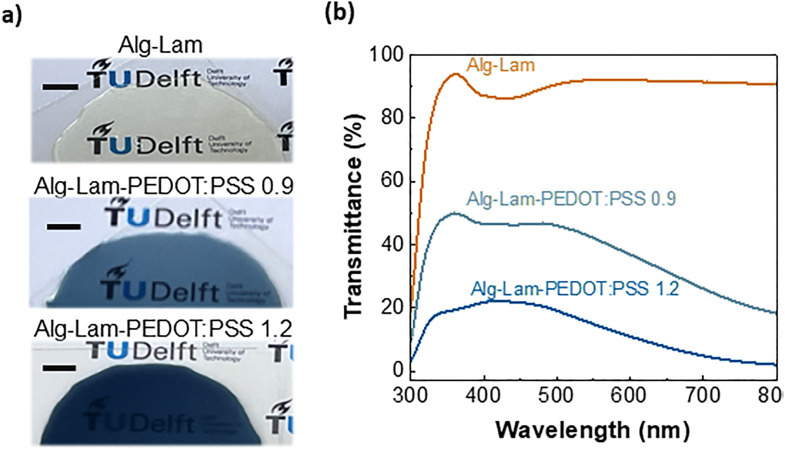
(a) Digital pictures of the different hydrogels studied with a thickness of 400 µm. Scale bars = 1 mm. (b) Optical transmittance of the Alg–Lam (orange line), Alg–Lam-PEDOT:PSS 0.9 (light blue line), Alg–Lam-PEDOT:PSS 1.2 (blue line) hydrogels with a thickness of 400 µm.

To evaluate the properties of the hydrogels we performed a number of characterization techniques. We focused on hydrogels with a controlled thickness of 400 µm made with two different concentrations of PEDOT:PSS – *i.e.* 0.9 and 1.2 wt%, referred to as Alg–Lam-PEDOT:PSS 0.9 and Alg–Lam-PEDOT:PSS 1.2, respectively. For comparison we used pure alginate–laminin hydrogels, referred to as Alg–Lam. First, we focused on the optical properties of the hydrogels and the results are shown in Fig. 2. Transparency is crucial for *in vitro* neural cultures since it allows high resolution microscopy to be performed. The digital pictures of all the formulations studied are shown in Fig. 2a. Although the inclusion of PEDOT:PSS in the hydrogels reduces transparency in the wavelength range of 300–800 cm^−1^, the Alg–Lam-PEDOT:PSS 0.9 maintains good transparency in the visible spectrum – a useful property for high resolution microscopy of neural cultures. These results were quantified *via* optical transmittance measurements as shown in Fig. 2b. The transparency of the Alg–Lam-PEDOT:PSS 0.9 hydrogel at *λ* = 500 nm was found to be 45.95%, and 19% for the Alg–Lam-PEDOT:PSS 1.2 hydrogel. These transmittance values can be controlled by tuning the thickness of the hydrogels, as shown in Fig. S1. We found that the transmittance of Alg–Lam-PEDOT:PSS 0.9 can be increased to 64%, at *λ* = 500 nm, by reducing the thickness from 400 µm to 150 µm. Similarly, the transmittance of the Alg–Lam-PEDOT:PSS 1.2 hydrogel can be increased to 35%. These results show that highly transparent electrically active hydrogels can be achieved despite the inclusion of the conducting polymer, which can be beneficial for *in vitro* neuron culture studies.

**Fig. 3:**
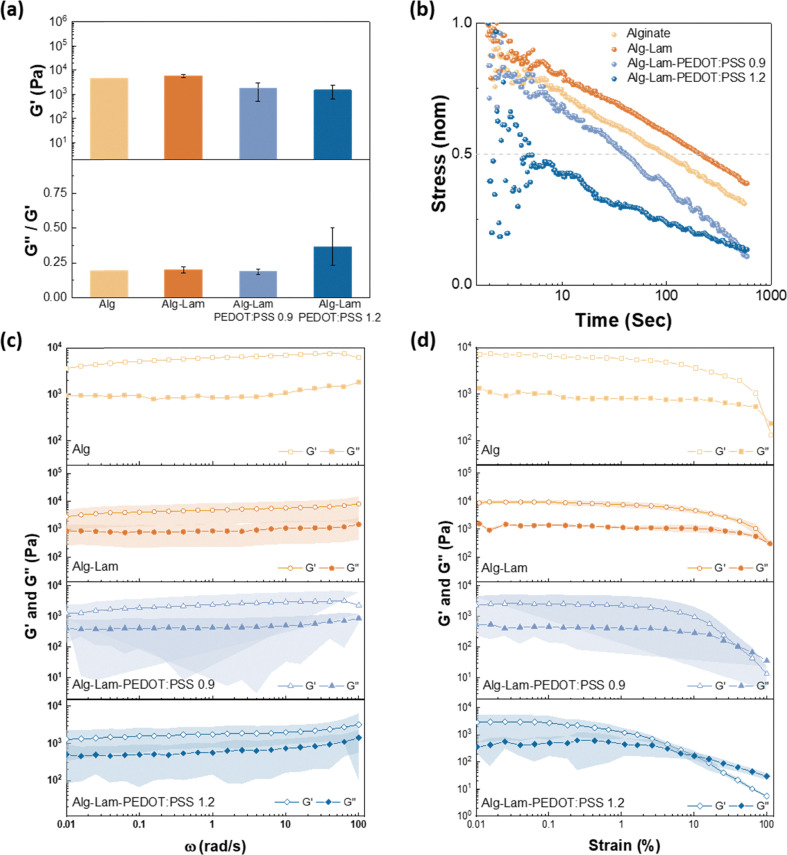
(a) The storage modulus (*G*′) and the damping factor (*i.e.* tan(*δ*) = *G*″/*G*′) measured at strain 0.5% at *ω* = 1 Hz. (b) Stress relaxation profiles for all the hydrogels developed in this study. (c) Frequency sweep from 0.01–100 rad s^−1^ at 0.05% strain and (d) strain sweep from 0.01 to 100% at 1 rad s^−1^.

Next we moved on to the characterization of the viscoelastic properties of the hydrogels – an important aspect for highly biomimetic neural cultures due to the viscoelastic properties of neural tissue.^30,31^ These properties were evaluated with oscillatory rheology and the results are shown in Fig. 3. Oscillatory time sweep tests were first applied to verify the gelation of alginate, Alg–Lam and Alg–Lam-PEDOT:PSS hydrogels. Due to the fast gelation of the alginate network, the crossover points were difficult to observe (Fig. S2). However, as shown in Fig. S2, the storage modulus (*G*′) was consistently higher than the loss modulus (*G*″), which proved the formation of the hydrogels. There was no significant change in *G*′ and *G*″ over the measurement period, indicating complete gelation stability for all compositions of the material and the final values are shown in Fig. 3a. The alginate only hydrogel showed an average *G*′ value of 4.59 kPa at a fixed frequency of 1 Hz, similar to Alg–Lam hydrogels (*i.e.* 5.74 ± 0.92 kPa). However, we found that *G*′ is reduced when PEDOT:PSS particles are incorporated in the hydrogel matrix, with Alg–Lam-PEDOT:PSS 0.9 measured at 1.74 ± 1.22 kPa and Alg–Lam-PEDOT:PSS 1.2 measured at 1.48 ± 0.83 kPa. To further evaluate the degree of solid-like response of the hydrogels, we also evaluated the damping factor as shown in the Fig. 3a bottom panel (*i.e. G*′/*G*″ or often found in the literature as tan(*δ*)). We found that for all of our hydrogel formulations the damping factor remained within a range of 0.2 to 0.4, with a slight increase for the hydrogels that include PEDOT:PSS. This proves that the viscoelastic properties of the material are dominated by the alginate network and slightly influenced by the inclusion of PEDOT:PSS.

The viscoelastic nature of the hydrogels is further evaluated with stress relaxation measurements as shown in Fig. 3b, which is a critical property for interfacing hydrogels with stem cell cultures.^32^ Stress relaxation reflects how quickly internal stresses in a material dissipate upon applying a fixed deformation; the relaxation half-time is the time to reach 50% of the initial stress. The impact of the hydrogel's stress relaxation profile on cells is context-dependent. In various 3D culture systems, faster-relaxing hydrogels allow cells to remodel their surroundings more readily and are typically associated with increased spreading and migration when other cues (*e.g.*, elastic modulus and ligand density) are held constant.^33,34^ As shown in Fig. 3b, the half-time relaxation is reduced when PEDOT:PSS particles are included in the hydrogel's matrix. Sulfonate groups have been reported to bind strongly to sodium alginate.^35^ Accordingly, we propose that the observed reduction in stress relaxation with an increase in PEDOT:PSS concentration likely results from the disruption of the Ca^2+^-mediated alginate network by the sulfonate-rich PSS phase. This disruption, likely due to competition for Ca^2+^ ions and electrostatic screening, decreases the effective crosslink density of the hydrogel network and facilitates accelerated stress relaxation.

As shown in Fig. 3c, all hydrogel compositions studied showed *G*′ and *G*″ values that were mostly constant across a range of frequencies (1–100 rad s^−1^) – indicating frequency independent solid-like behavior. The differences in yielding behavior across compositions were also examined and the measurements are shown in Fig. 3d. The yield strain was extracted by identifying the crossover point of *G*′ and *G*″ (from Fig. 3d). Yield strain is the critical strain at which the material transitions from elastic (reversible deformation) to plastic (irreversible deformation) behavior. For hydrogel matrices employed for cell culture, a lower yield strain implies that plastic remodeling initiates at smaller deformations, which can facilitate cell extension and network reorganization when other cues (*e.g.*, modulus and ligand density) are held constant. Conversely, lower yield strain typical indicates inferior mechanical stability for long-term structural retention of the hydrogel, emphasizing that the preferred yield strain is context depended. We found that the inclusion of PEDOT:PSS has a significant effect on the yield strain of the hydrogels which was found at 91.74%, 110.44 ± 7.86%, 35.19 ± 13.44%, and 11.53 ± 7.01% and for Alg, Alg–Lam, Alg–Lam-PEDOT:PSS 0.9 and Alg–Lam-PEDOT:PSS 1.2, respectively. We propose that the observed reduction in yield strain is likely due to competition for Ca^2+^ ions and electrostatic screening of PSS^−^ sulfonate groups in PEDOT:PSS particles. Moreover, the PEDOT:PSS particles embedded within the hydrogel matrices do not form strong inter-particles crosslinks even above their percolation threshold. Therefore, PEDOT:PSS domains within the hydrogels likely act as compliant, weakly coupled inclusions that facilitate interfacial slip and early yielding under increasing strain levels.

**Fig. 4:**
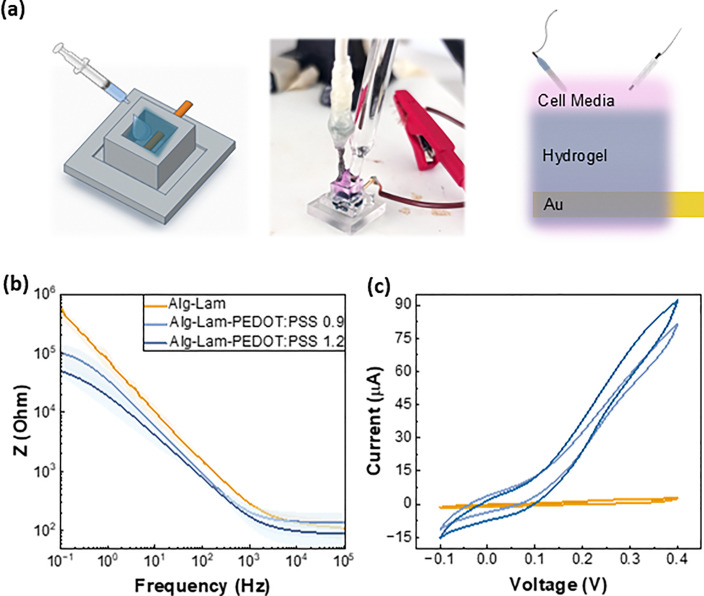
(a) Schematic and digital picture of the electrochemical setup developed to probe the properties of the hydrogels and the suggested equivalent circuit. A custom made 3D printed mold (*L* = 5 mm × *W* = 5 mm × *H* = 10 mm) with an integrated gold pogo pin encapsulated in the hydrogels of 1 cm thicknesses. All measurements were performed in cell culture media (DMEM/F12 – pH 7.4) at 37 °C, with the support of an Ag/AgCl reference and a Pt mesh counter electrode immersed fully in the electrolyte. (b) The impedance magnitude *versus* frequency plots obtained with electrochemical impedance measurements for Alg–Lam (orange lines), Alg–Lam-PEDOT:PSS-0.9 (light blue lines) and Alg–Lam-PEDOT:PSS-1.2 (blue lines). 3 samples were measured in each case and the statistical error bars are represented with the shaded areas around each line. (c) Cyclic voltammograms of the different hydrogels studied, obtained with a scan rate of 0.1 V s^−1^.

Optical transmittance and oscillatory rheology measurements proved that the hydrogels can be rendered transparent, soft, and viscoelastic – important parameters for neuron cultures. The electrochemical characterization of hydrogels was investigated with electrochemical impedance spectroscopy (EIS) and cyclic voltammetry (CV), using a custom made setup shown in Fig. 4a, and Fig. S3. 3D printed molds with fixed dimensions (*i.e.* length = 5 mm × width = 5 mm × height = 10 mm) were used and the hydrogels were directly casted in, with a controlled thicknesses of 10 mm. An integrated gold pogo pin allows for stable gold/hydrogel contacts and accurate evaluation of the electrochemical operation of the hydrogels, with reliable results across samples. Our initial trials to measure the electrochemical properties of the hydrogels by simply casting the hydrogels on gold or indium-tin-oxide flat substrates did not yield reliable results due to the weak adhesion of the soft hydrogels on the rigid electrodes, resulting in delamination and inconsistent readings (Fig. S4). In our approach, we kept all the parameters of the measurements constant, with the only changing parameter being the PEDOT:PSS concentration in the hydrogels. The samples were immersed in cell media overnight to ensure homogeneity in swelling and reference (Ag/AgCl) and counter (Pt mesh) electrodes were used to measure EIS and CV. The cell culture medium used was Dulbecco's modified Eagle's medium (DMEM), containing amino acids, vitamins, and glucose, and maintained at a physiologically relevant pH of 7.4. The same medium was employed during hydrogel formation, and its use in electrochemical measurements ensured the stability of the hydrogel system by minimizing osmotic effects arising from differences in concentration and composition between the hydrogel and the cell culture environment. Such a system is highly customizable and allows for measurements under consistent hydration conditions.

The impedance magnitude over a range of frequency (*i.e.* 10^5^–0.1 Hz) of the hydrogels was extracted from EIS measurements and is shown in Fig. 4b, and the mixed ionic electronic conduction properties of the hydrogels can be evaluated by looking at the different frequency bands of the impedance. As recently suggested by Daso *et al.*,^36^ the impedance spectra of a hydrogel system with electrically conducting nanoparticle fillers can be simulated with an equivalent circuit to distinguish ionic and electronic conductivities. As it is also reported elsewhere, the high frequency band (>10^4^ Hz) is dominated by the fast ionic movement in the hydrogels and low frequency band (<10 Hz) is dominated by the electronic conductivity and the interaction of the electronic current with the ionic current within the hydrogels.^37^ Therefore, we were able to extract the ionic and electronic conductivities of the different hydrogel systems by simply using the resistance values at *f* = 10^5^ Hz and at 0.1 Hz, respectively from the resistance (*i.e.* real part of impedance) *versus* frequency plot in Fig. S5. The ionic and electronic conductivities were then extracted using eqn (2) (see the Experimental section). No significant changes in the ionic conductivity values were observed for the different systems under study – *i.e.* ionic conductivity of 1.6 × 10^−4^, 1.3 × 10^−4^, and 2 × 10^−4^ S m^−1^ for the Alg–Lam, Alg–Lam-PEDOT:PSS 0.9, and Alg–Lam-PEDOT:PSS 1.2, respectively. However the electronic conductivity of hydrogels loaded with PEDOT:PSS increases by approximately 10 times compared with the hydrogels with no PEDOT:PSS loading, *i.e.* 2.9 × 10^−4^; 1.9 × 10^−3^; 4.1 × 10^−3^ S m^−1^ the Alg–Lam, Alg–Lam-PEDOT:PSS 0.9, and Alg–Lam-PEDOT:PSS 1.2, respectively. These results suggest that our hydrogel system comprises PEDOT:PSS particles stably embedded within the hydrogel matrix, with partially developed percolation pathways that enable electron conduction. These percolation pathways appear to be better interconnected in Alg–Lam-PEDOT:PSS 1.2 compared to Alg–Lam-PEDOT:PSS 0.9, as evidenced by a slight increase in electronic conductivity from 1.9 to 4.1 × 10^−3^ S m^−1^. However, this increase was not statistically significant, as variations were observed across samples, as shown by the shaded regions around each main line in Fig. 4b for the hydrogels studied. The statistical error bars were calculated from five samples in each case, and similar trends were confirmed in multiple independent experimental batches. These findings suggest that the percolation network is already well established at a 0.9 wt% PEDOT:PSS loading, and that a further increase of 0.3 wt% provides limited enhancement in pathway development. Interestingly, as observed in Fig. 2, increasing the loading to 1.2 wt% notably reduces the transparency of the hydrogels while providing minimal gains in electronic conductivity—an important design consideration for subsequent cell culture studies. This hypothesis and interpretation are illustrated schematically in Fig. 1.

Importantly, we were able to also clearly observe a capacitance increase for the hydrogels loaded with PEDOT:PSS compared with the pure Alg–Lam hydrogel. From the capacitance *vs.* frequency plots in Fig. S5, we found that the value of the capacitance at 0.1 Hz is at 2.9 × 10^−6^, 4.2 × 10^−5^, and 7.5 × 10^−5^ F for the Alg–Lam, Alg–Lam-PEDOT:PSS 0.9, and Alg–Lam-PEDOT:PSS 1.2, respectively. This represents more than 10 times increase when 1.2 wt% of PEDOT:PSS is loaded in the pure Alg–Lam hydrogel network. These results show that PEDOT:PSS particles can attract charge and render the hydrogel electrically active, even without fully developed percolation networks for optimum electronic conductivity.

The capacitive properties of the hydrogel loaded with PEDOT:PSS were further verified with cyclic voltammetry, as shown in Fig. 4c. By integrating the graph area enclosed by the CV for each of the hydrogel systems, and applying eqn (3) (see the Experimental section) we calculated the capacitance values to be 5.8 × 10^−6^, 3.4 × 10^−5^ and 5.2 × 10^−5^ F for Alg–Lam, Alg–Lam-PEDOT:PSS 0.9 and Alg–Lam-PEDOT:PSS 1.2, respectively. Here we note that the scan rate we use for the CV measurements (*i.e.* 0.1 V s^−1^) corresponds to an equivalent frequency of 0.2 Hz, according to eqn (4) (see the Experimental section), and therefore the values of capacitance are in line with the capacitance extracted from the EIS at 0.1 Hz, and verify the accuracy of our measurements and our analysis. We have also studied CVs at different scan rates as shown in Fig. S6, where we observe increased capacitance of the hydrogels that include PEDOT:PSS at all scan rates studied, verifying the stable inclusion of the electroactive particle of PEDOT:PSS within the alginate/laminin hydrogel network. We were able to calculate the volumetric capacitance (C*) using the capacitance values extracted from Fig. 4c. The fixed sample volume was accurately defined by the dimensions of the 3D-printed molds and the reproducible hydrogel thickness (*i.e.* 0.25 cm^3^). Based on these parameters, the calculated C* values for the various hydrogel systems were 2.32 × 10^−5^, 1.36 × 10^−4^, and 2.07 × 10^−4^ F cm^−3^, respectively. All the properties of the different hydrogels under study are summarized in Table 1.

**Table 1:** Summary of the key properties measured for the hydrogels developed in this study

	Transmittance at *λ* = 500 nm (%) *d* = 400 µm (%)	*G*′ (kPa) *d* = 500 µm	*G*″/*G*′ *d* = 500 µm	Electronic conductivity-EIS@0.1 Hz S m^−1^*d* = 10 mm	Capacitance–EIS@0.1 Hz (F) *d* = 10 mm	Capacitance–CV (F) *d* = 10 mm	C* − CV (F cm^−3^)
Alg–Lam	90	5.74	0.2	2.9 × 10^−4^	2.9 × 10^−6^	5.8 × 10^−6^	2.32 × 10^−5^
Alg–Lam-PEDOT:PSS 0.9	46	1.74	0.19	1.9 × 10^−3^	4.2 × 10^−5^	3.4 × 10^−5^	1.36 × 10^−4^
Alg–Lam-PEDOT:PSS 1.2	19	1.48	0.37	4.1 × 10^−3^	7.5 × 10^−5^	5.2 × 10^−5^	2.07 × 10^−4^

The detailed optical, mechanical and electrochemical characterization showed promising results for interfacing the electrically active hydrogels with neural tissue. To further evaluate the potential of the materials for neural cultures we performed a series of experiments. First, we measured the changes in pH in the cell media after incubating the hydrogels for over 48 hours, to assess potential release of acidic PEDOT:PSS particles. The pH was measured at 7.82 when incubated with Alg–Lam-PEDOT:PSS hydrogels for 48 hours, compared to pH 7.77 in the control media. These results were verified with 5 samples in each case, and indicate that no PEDOT:PSS particles are leaching out of the hydrogels.

To further support these observations, we found that the electrically active hydrogels preserved their shape and size over four weeks when immersed in cell media and exposed in a cell incubator (Fig. S8). We note that different concentrations of PEDOT:PSS were used for these experiments, ranging from 0.3 to 1.2 wt%, with identical results obtained in all cases. All cell culture data presented in this study were obtained with an electrically active hydrogel with 0.6 wt% concentration of PEDOT:PSS loaded in the hydrogel matrix. The electrochemical properties of electrically active hydrogels loaded with 0.6 wt% PEDOT:PSS were similar to the hydrogels loaded with 0.9 wt% PEDOT:PSS, as shown in Fig. S9. To further evaluate the cytocompatibility of the electrically active hydrogels, we used SH-SY5Y cell cultures growing alongside the different hydrogel formulations – a model neuroblastoma cell line that is widely used in *in vitro* neural culture studies.^38^ As shown in Fig. S9 after 7 days *in vitro* the cells adhere, proliferate and start differentiating on all well plates regardless of the concentration of PEDOT:PSS. Live/dead assays revealed that the cell viability was over 90% in all cases (Fig. S9), proving the non-cytotoxic nature of the hydrogels.

**Fig. 5:**
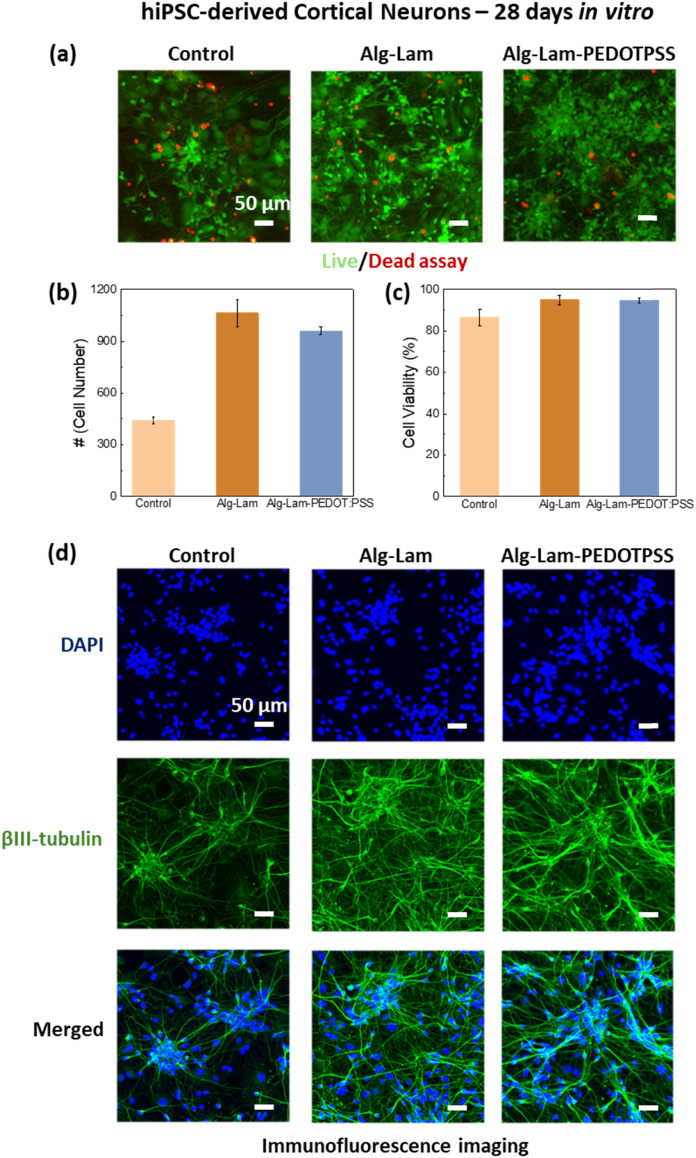
(a) Live/Dead staining of hiPSC-derived cortical neurons on day 28 *in vitro*. From left to right – control cultures grown on polystyrene well plates, on polystyrene well plates together with Alg–Lam hydrogels and on polystyrene well plates together with Alg–Lam-PEDOT:PSS hydrogels. (b) Average live cell count and (c) percentage of cell viability of hiPSC-derived cortical neurons directly exposed to the alginate–laminin and alginate–laminin-PEDOT:PSS hydrogels. (d) Immunofluorescence imaging of hiPSC-derived cortical neurons cultures stained with the nuclear marker DAPI (blue) and the neuronal maker βIII-tubulin (green) on day 28 *in vitro*. Scale bars = 50 µm.

Lastly, we have incubated our electrically active hydrogels with cortical neurons derived from human induced pluripotent stem cells (hiPSCs). The hydrogels are stable and maintain their shape and integrity (Fig. S10) during the whole duration of the neuron differentiation and maturation timeline which spans over 28 days as shown in Fig. 5. Live/dead assays performed to the cell cultures growing alongside the hydrogels at day 28, revealed excellent cell viability for the cells as shown in Fig. 5(b–d). We calculated 86.43%, 94.98% and 94.74% cortical neuron viability when cultured under controlled conditions, alongside the Alg–Lam hydrogel and the Alg–Lam-PEDOT:PSS hydrogel, respectively. These results clearly show that our proposed electrically active hydrogels are biocompatible and suitable for long term hiPSC differentiation protocols. To better identify the quality of our cortical neural culture, we performed immunofluorescence imaging on cultures at day 28 *in vitro*. All cultures were stained with the nuclei marker DAPI (blue) and the neuronal marker βIII-tubulin (green), as shown in Fig. 5. Both the cortical neurons that have been matured alongside the hydrogels with and without PEDOT:PSS show comparable cell density, neurite population and network formation, showing that the PEDOT:PSS inclusion in the hydrogels do not disrupt the delicate processes of neuronal differentiation and maturation. Here, it is worth noting that our proposed hydrogels are designed for future 3D encapsulation of hiPSCs and the development of 3D neural networks. The highly tunable nature of the electrical activity of our designed hydrogel system will allow for a fundamental understanding of the effects of electrical cues on 3D stem cell growth into functional neural networks. However, it is well established that alginate-based hydrogels must be functionalized with the arginine–glycine–aspartate (RGD) peptide, as cells do not adhere or proliferate in its absence.^39^ Because our alginate system was not functionalized with RGD, establishing 3D cell encapsulation protocols was not feasible. Nevertheless, our results clearly demonstrate the structural integrity and cytocompatibility of the proposed electrically active hydrogels for long-term hiPSC-derived neural cultures, highlighting their potential for future 3D neural network development upon alginate functionalization.

## Conclusion

We demonstrated the fabrication of electrically active hydrogels tailored for *in vitro* neural cultures, by integrating a viscoelastic alginate matrix interpenetrated with the neural protein laminin and embedded with conductive PEDOT:PSS particles. The electrically active hydrogels were prepared by mixing commercially available PEDOT:PSS dispersions, without any modification, with alginate and laminin solutions and casting them in custom 3D-printed molds, where slow Ca^2+^ release from CaSO_4_ ensured uniform gelation and precise control over the hydrogel thickness. Detailed optical, mechanical, and electrochemical analyses revealed a combination of properties highly suited for neural cell cultures, supporting the use of these electrically active hydrogels as ideal materials for future biohybrid electronic interfacing of neural tissue. Optical transmittance measurements demonstrated good transparency across the visible spectrum, which can be finely tuned by adjusting the PEDOT:PSS concentration and hydrogel thickness. Transmittance values exceeding 60% at *λ* = 500 nm were achieved for 150 µm thick hydrogels. This is a key feature for high-resolution optical microscopy assessment of neural cultures, which is often overlooked in the design of similar biohybrid systems. In addition, we demonstrated that the hydrogels exhibit suitable mechanical properties for neural interfacing, with storage and loss moduli in the range of 1–10 kPa. Oscillatory rheology measurements and analyses showed that hydrogels containing PEDOT:PSS particles show shorter relaxation half-times, suggesting a more dynamically permissive matrix for neural remodelling. Importantly, we developed an electrochemical setup to reproducibly characterize the electronic and ionic conduction within the electrically active hydrogels. Electrochemical impedance and cyclic voltammetry measurements showed that the inclusion of PEDOT:PSS within the hydrogel matrix enhanced electrical conductivity and charge storage capacitance, with good operational stability over 80 electrochemical cycles. These results show that PEDOT:PSS particles are stably integrated within the hydrogel matrix and can form partial percolation pathways to attract charge and render the hydrogel electrically active. We examined PEDOT:PSS loadings from 0.6 to 1.2 wt%, representing the maximum concentration attainable from commercial dispersions without modification. While higher loadings slightly improved electronic conductivity and charge storage capacitance, they also caused a substantial loss in optical transparency, underscoring the need to balance electrical and optical performance in bioelectronic hydrogel systems. Finally, we found that the proposed electrically active hydrogels are highly compatible with cell cultures. The hydrogels demonstrated excellent stability under cell culture conditions for more than 28 days when maintained with human induced pluripotent stem cell-derived cortical neurons. As demonstrated by live/dead assays, the cultures show no distinct differences between the different hydrogel systems, indicating the highly cytocompatible nature of the proposed electrically active hydrogels.

To conclude, we developed electrically active hydrogel systems that combine desirable optical, mechanical, electrical, and cytocompatible properties for stem cell cultures. The tuneable nature of this system enables its future use as an electrically active scaffold to support and guide the growth of highly biomimetic three-dimensional neural cultures derived from human stem cells. Our findings position electrically active hydrogels based on PEDOT:PSS as versatile biohybrid platforms for next-generation bioelectronic neural systems, with potential applications in both *in vitro* studies and *in vivo* bioelectronic interfaces.

## Experimental

### Materials

PEDOT:PSS (Clevios PH 100) was purchased from Heraeus, Germany. Sodium alginate (PRONOVA® UP VLVG, product NO. 42000001) with a molecular weight (Mw) less than 75 kDa, Dulbecco's modified Eagle's medium (10×, High glucose, product NO. D2429), and calcium sulfate dihydrate (ACS reagent, 98%, product NO. 255548) were used. Laminin from the Engelbreth–Holm–Swarm murine sarcoma basement membrane (product NO. L2020) was purchased from Sigma-Aldrich. Laminin (red fluorescent, rhodamine, Cat.#LMN01-A) was purchased from Cytoskeleton, Inc.

### Conductive hydrogel preparation

All hydrogel samples were based on 2% w/v alginate hydrogels. Pure alginate hydrogels were prepared by mixing 2.5% w/v sodium alginate and 183 mM CaSO_4_ diluted in 1× Dulbecco's modified Eagle's medium (DMEM). 0.8 mL of 2.5% w/v sodium alginate and 0.2 mL of 183 mM CaSO_4_ were loaded into two separate syringes, and connected with a female–female Luer-lock connector. The solutions were mixed rapidly by pushing the syringes for approximately 6 times. Due to the fast gelation, the mixed solution was dropcast in 3D printed molds with controlled thickness and shape, and left for one hour to complete gelation. Alginate/PEDOT:PSS/laminin hydrogels were prepared by mixing 22.5 mg of sodium alginate and 563 µL of PEDOT:PSS with 90 µL of 10X DMEM and 237 µL of MiliQ H_2_O. The mixture was left stirring overnight to ensure homogeneous mixing. 10 µL of laminin was then added into the mixture and gently stirred in an ice bath to avoid laminin gelation. The solution was then loaded in a syringe and the hydrogels were cast using the same method as the pure alginate hydrogels.

### Viscoelastic properties by oscillatory rheology

The mechanical properties of the hydrogels were investigated with an AR 2000 rheometer. According to the hydrogel preparation method mentioned above, the hydrogel precursor was well-mixed with the calcium sulphate dispersion and directly loaded onto the Peltier plate (8 mm in diameter) of the rheometer for rheological analysis. After sample loading, the gap height was adjusted to 0.5 mm, and after gelation, the temperature for all the measurements was maintained at 37 °C to simulate the cell culture condition. Strain sweeps were performed from 0.01% to 100% to determine the yield point and appropriate value setting for strain (Fig. S2). The value of applied strain should be chosen before the yield point is reached, and should also fulfill the premise of being able to apply sufficient stimulation to the samples. Here we select the value as 0.5%. Oscillation–time sweep under 0.5% strain and 1 rad s^−1^ frequency was applied for 1 hour until the gelation process was completed. Next, an extra 15-minute oscillation–time sweep was performed to use the last set of data points as the storage modulus and loss modulus of the material. The dumping factor tan *δ* can be calculated using the following equation:1
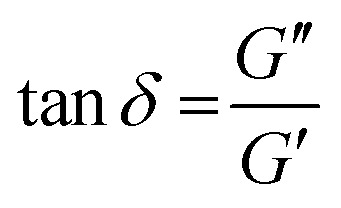
where *G*′ is the storage modulus and *G*″ is the loss modulus.

Stress relaxation tests were measured first under 1% strain with a duration of 600 s and then under 1% strain for another 600 s.

### Optical transmittance measurements

To ensure consistent results, all the samples for transmittance measurements were prepared by drop-casting them onto glass substrates, which were controlled to thicknesses of 150, 400, and 1000 µm with 3D-printed spacers. Transmittance was measured using a LAMBDA UV/vis spectrophotometer (PerkinElmer), with the incident light wavelengths ranging from 300 to 800 nm.

### Electrochemical characterization

All the measurements were performed with a PalmSens4 potentiostat at 37 °C in cell media. The experimental setup followed the standard three-electrode setup by using gold pogo pins (diameter = 1.13 mm) integrated within a homemade 3D-printed mold with dimensions of width = 5 mm × length = 5 mm × height = 10 mm (Fig. S3) as working electrodes, a mesh Pt electrode (diameter = 0.06 mm, open area = 65%, 20 × 20 mm) as the counter electrode and Ag/AgCl as the reference electrode. Prior to each measurement, all the samples were incubated in DMEM/F12 cell culture media and placed in the fridge to rest overnight. Electrochemical impedance spectroscopy (EIS) measurements were performed within the frequency range of 0.1–10^5^ Hz with an AC voltage perturbation of 10 mV at 0 V *vs.* the reference electrode. Cyclic voltammetry (CV) measurements were performed from −0.1 to 0.4 V at different scan rates 0.1 V s^−1^, 0.2 V s^−1^ and 0.5 V s^−1^. All the results presented here are extracted from at least three replicates for each condition and verified in many more experimental batches. The conductivity of the different systems studied was calculated by converting the resistance at 10^5^ Hz to conductivity using the following equation:^37^2
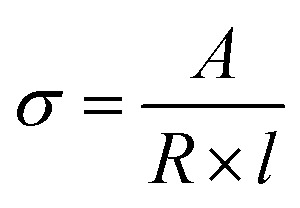
where *A* is the electrode surface (*i.e.* gold pogo pin diameter = 1.13 mm × π × length = 5 mm), *l* is the distance between working electrode and the counter electrode (*i.e.* 10 mm) and *R* is the resistance value extracted from the real impedance *versus* frequency plot (Fig. S5) at different frequencies (*i.e.* 10^5^ Hz for ionic conductivity and 0.1 Hz for electronic conductivity). The capacitance values from EIS were extracted directly from the impedance values at 0.1 Hz. The capacitance from cyclic voltammograms was calculated by integrating the current over the full potential range from the following equation:3
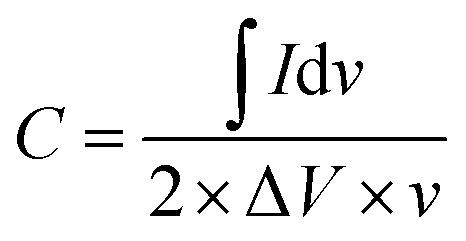
where 
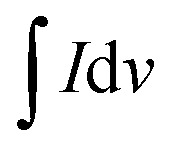
 is the polygon area of the cyclic voltammogram, Δ*V* is the potential window and *v* is the scan rate. The correlation between the scan rate of cyclic voltammograms and frequency was extracted from the following equation:4
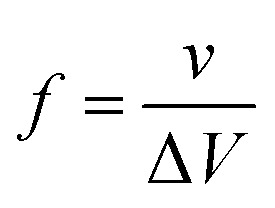
where *v* = the scan rate and Δ*V* is the potential window, which was constant at 0.5 V in all of our studies (*i.e.* −0.1 V to 0.4 V).

### SH-SY5Y human neuroblastoma-derived immature neuron-like cells

Prior to seeding onto the samples, human neuroblastoma SH-SY5Y cells (Sigma-Aldrich, #94030304) were maintained in Dulbecco's modified Eagle's medium/Nutrient Mixture F-12 (DMEM/F-12, 1 : 1; Thermo Fisher Scientific, #10565018) supplemented with 10% fetal bovine serum (FBS; Sigma-Aldrich, F7524) and 1% penicillin–streptomycin. Cells were cultured at 37 °C in a humidified atmosphere containing 5% CO_2_. Upon reaching confluence, cells were detached using Accutase for 5 min, followed by centrifugation at 900 rpm for 5 min. The resulting cell suspension was seeded onto 24-well plates at a density of 10 000 cells per cm^2^, with hydrogel samples (5 mm in diameter, 1 mm in thickness) placed in parallel wells, which were incubated with cell media the night before to ensure full swelling and absorption of cell media nutrients. After three days of culture, differentiation was induced by supplementing the media with 10 µM retinoic acid (Sigma-Aldrich, R2625) for an additional three days to promote the formation of immature neuron-like cells.

### hiPSC-derived cortical neuron culture

Human induced pluripotent stem cell (hiPSC)-derived neural progenitor cells (NPCs; line SCTi003-A, StemCell Technologies, Catalog #200-0620) were thawed and cultured on Matrigel-coated 6-well plates for 4 days to allow expansion. Prior to cell seeding, wells of a 24-well plate were sequentially coated with poly-d-lysine (PDL; 0.1 mg mL^−1^, 2 h at 37 °C; Gibco, #A38904-01) followed by laminin (100 µg mL^−1^, 2 h at 37 °C; Sigma-Aldrich, #L2020). NPCs were seeded at a density of 50 000 cells per well, with hydrogel samples (5 mm in diameter, 1 mm in thickness) placed in parallel wells. Differentiation into cortical neurons was induced for 7 days using a STEMdiff™ Midbrain Neuron Differentiation Kit (StemCell Technologies, #100-0038). Subsequently, hiPSC-derived cortical neurons were maintained and matured in BrainPhys™ hiPSC Neuron Kit complete media (StemCell Technologies, #05795) supplemented with SM1, N2, brain-derived neurotrophic factor (BDNF), glial cell line-derived neurotrophic factor (GDNF), ascorbic acid, and dibutyryl-cAMP, as provided in the kit. Cultures were maintained for the duration of the experiment with half-media changes every two days to promote neuronal maturation. All media were supplemented with 1% penicillin–streptomycin (Invitrogen, #15140122).

### Viability assay

Viability assays were performed using a LIVE/DEAD™ Cell Imaging Kit (488/570) from Thermo Fisher Scientific. Calcein AM, a cell permeant dye, was used as the live cell indicator (green) and BOBO-3 Iodide was used as the dead cell indicator (red). The viability assay was performed using 1 µM calcein AM and 1 µL of BOBO-3 Iodide in DMEM/F12 media. For both SH-SY5Y neuroblastoma cells and hiPSC-derived cortical neurons, the well plates were incubated for 15 min in a cell incubator with the reagents and respective culture media (DMEM/F12 media for the SH-SY5Y neuroblastoma cells and complete Brainphys media for the hiPSC-derived cortical neurons) and then washed with PBS (x1) before topping up again with cell culture media. The well plates were directly imaged using a Keyence BZ-810 microscope system (Osaka, Japan).

### Immunohistochemistry

At day *in vitro* 28 (DIV28), hiPSC-derived cortical neurons were fixed with 4% paraformaldehyde for 15 min at room temperature (RT) and subsequently permeabilized with 0.5% Triton X-100 (Sigma-Aldrich, St. Louis, MI, USA) in phosphate-buffered saline (PBS; Sigma-Aldrich) for 15 min. Following permeabilization, the cells were washed three times with PBS for 5 min each at RT. To prevent non-specific antibody binding, samples were incubated with bovine serum albumin (BSA; Sigma-Aldrich, #9048468) for 30 min at RT, followed by three additional PBS washes (5 min each). The cells were then incubated with the primary antibody, mouse monoclonal anti-βIII-tubulin (2G10-TB3; eBioscience™, #14-4510-82; 0.5 mg mL^−1^) and diluted 1 : 100 in 1% BSA in Dulbecco's PBS (dPBS) for 1 h at RT. After washing, the samples were incubated with the secondary antibody, goat anti-mouse Alexa Fluor 488 (Abcam, #ab150113) and diluted 1 : 500 in 1% BSA in dPBS, for 1 h at RT in the dark. Finally, cell nuclei were counterstained with NucBlue (Life Technologies, Carlsbad, CA, USA) for 20 min. Fluorescence images were acquired using a Keyence BZ-810 microscope system (Keyence, Osaka, Japan).

## Conflicts of interest

A patent application has been filed on the materials described in this work.

## Supplementary Material

TC-014-D5TC02708J-s001

## Data Availability

The data supporting this article have been included as part of the supplementary information (SI). Supplementary information is available. See DOI: https://doi.org/10.1039/d5tc02708j.
