# Supplementary material for: Electrically active hydrogels based on PEDOT:PSS for neural cultures
Source: J Mater Chem C Mater. 2025 Oct 28;14(2):648–59. doi: 10.1039/d5tc02708j (PMC12620795; doi:10.1039/d5tc02708j)
Supplement: TC-014-D5TC02708J-s001 [file TC-014-D5TC02708J-s001.pdf]

Supplementary Information (SI) for Journal of Materials Chemistry C.
This journal is © The Royal Society of Chemistry 2025

Supporting Information

Electrically Active Hydrogels Based on PEDOT:PSS for Neural Cultures

Liwen Wang[1], Yannick Hajee[2], Jean-Philippe Frimat[3], Mani Diba[2], Achilleas Savva[1]*

[1]Department of Microelectronics, Faculty of Electrical Engineering, Computer Science and Mathematics, Delft University of Technology, Delft, the Netherlands

[2] Regenerative Biomaterials–Dentistry, Research Institute for Medical Innovation, Radboud University Medical Center, Nijmegen, the Netherlands

[3]Department of Human Genetics, Leiden University Medical Center, Leiden, the Netherlands

*E-mail: a.savva@tudelft.nl

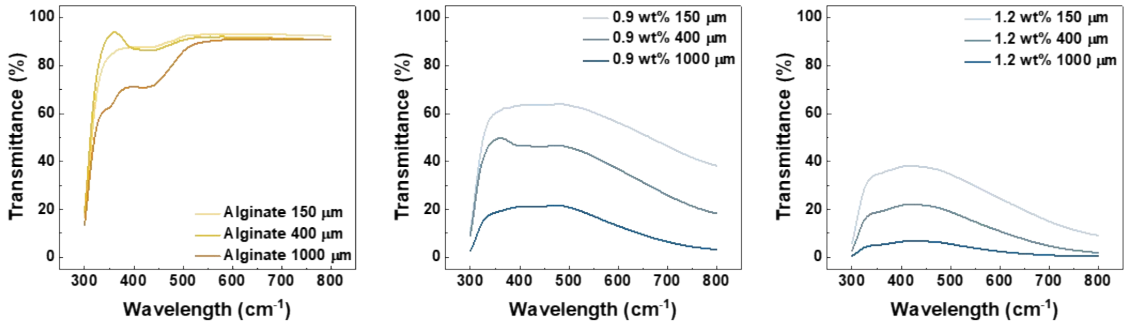

Figure S1: Optical transmittance measurements for hydrogels with 0, 0.9 and 1.2 wt% PEDOT:PSS loading with different thicknesses – i.e. 150, 400 and 1000 µm.

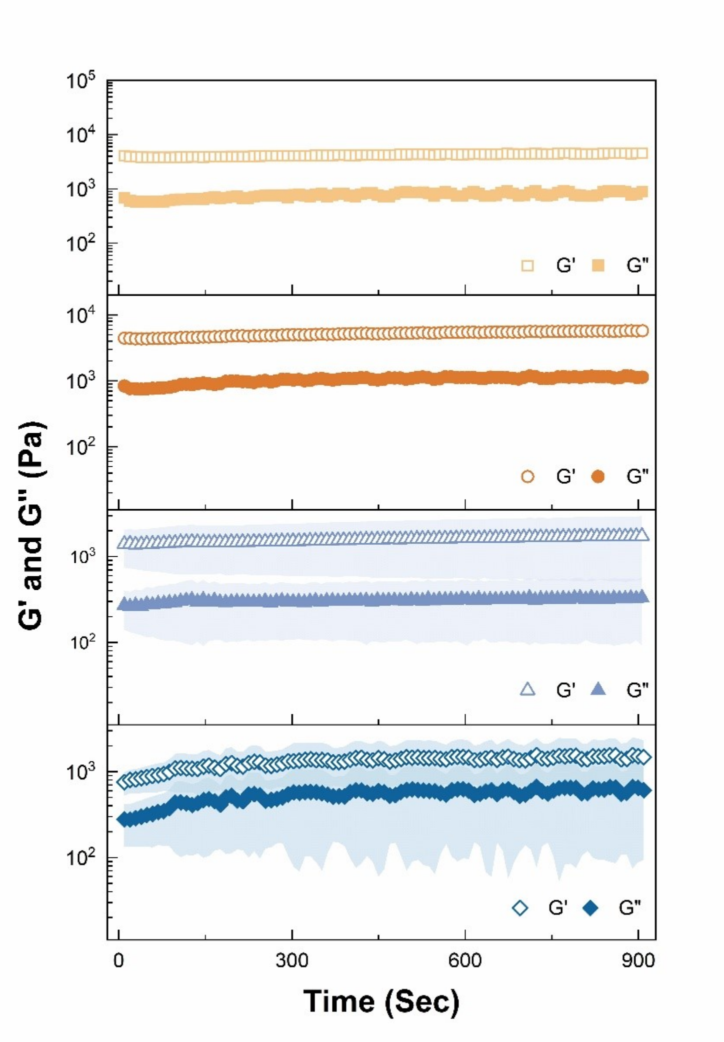

Figure S2: Time sweep for all the hydrogels studied for more than one hour gelation.

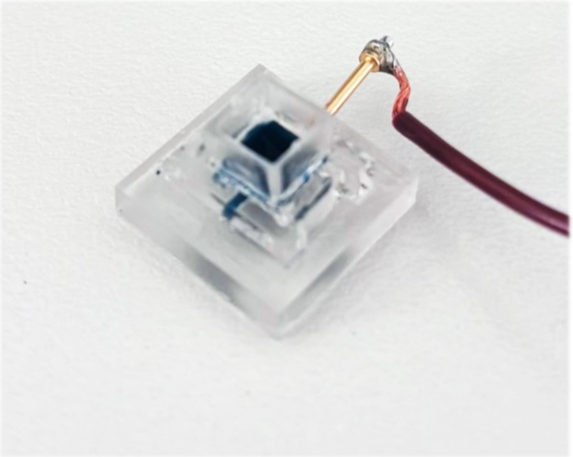

<u>Figure S3</u>: Homemade electrochemical measurement set-up, used to accurately measure electrochemical impedance spectroscopy and cyclic voltammetry.

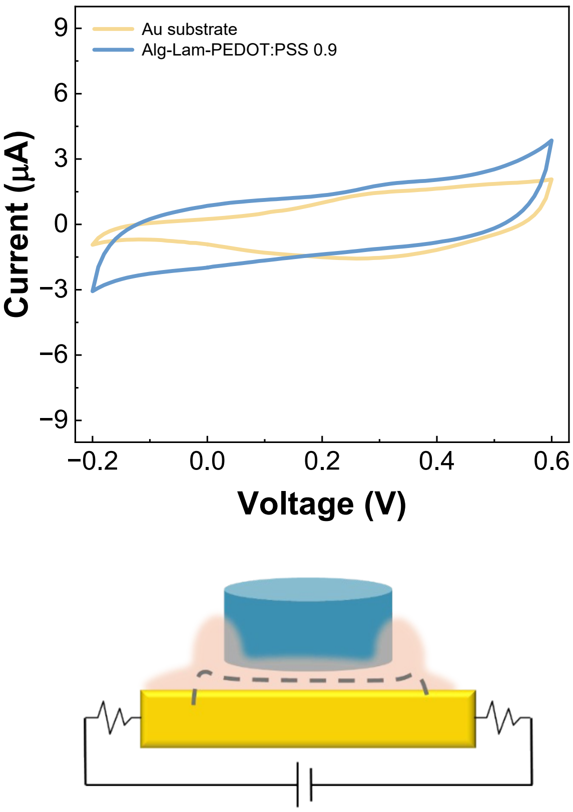

<u>Figure S4</u>: Cyclic voltammogramms (top) obtained from hydrogels casted on gold coated glass substrates. The measurements are unreliable due to poor contact between the hydrogel sample and the conductive substrate, caused by water penetration at the interface and subsequent hydrogel detachment (bottom schematic).

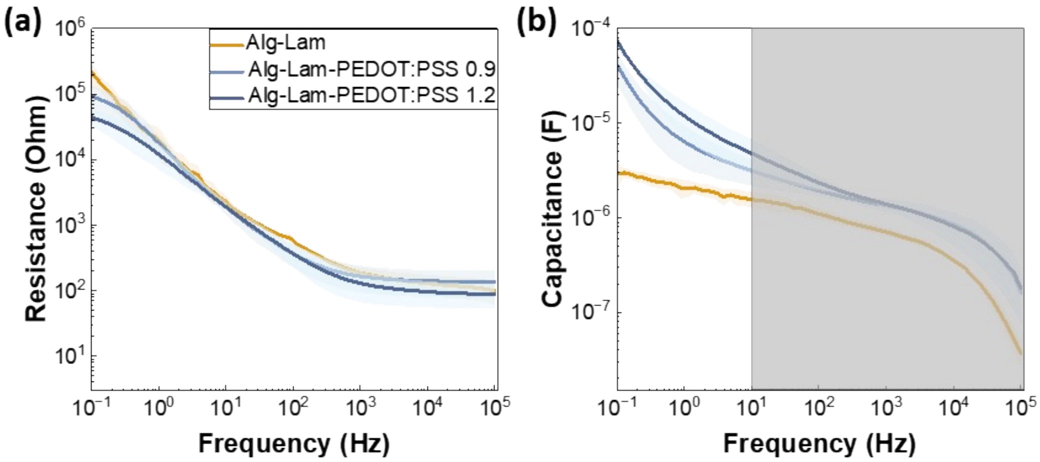

<u>Figure S5</u>: The **a)** resistance (i.e. real impedance and **b)** capacitance versus frquency plots obtained from EIS measurements.

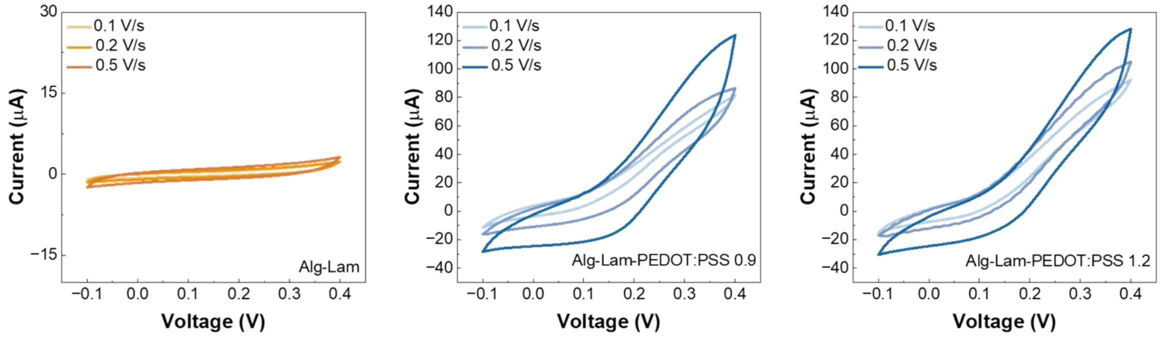

<u>Figure S6</u>: Cyclic voltammograms obtained using the setup shown in figure S3, for the different hydrogels under study for different scan rates 0.1, 0.2 and 0.5 V/s.

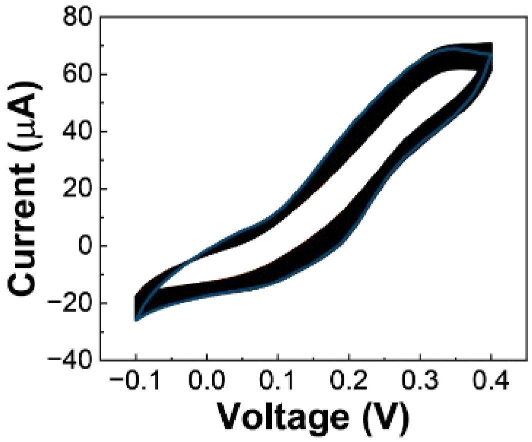

<u>Figure S7</u>: Cyclic voltammogramms collected after 80 cycles, using the setup decribed in figure S3 at a scan rate of 0.1 V/s in cell media.

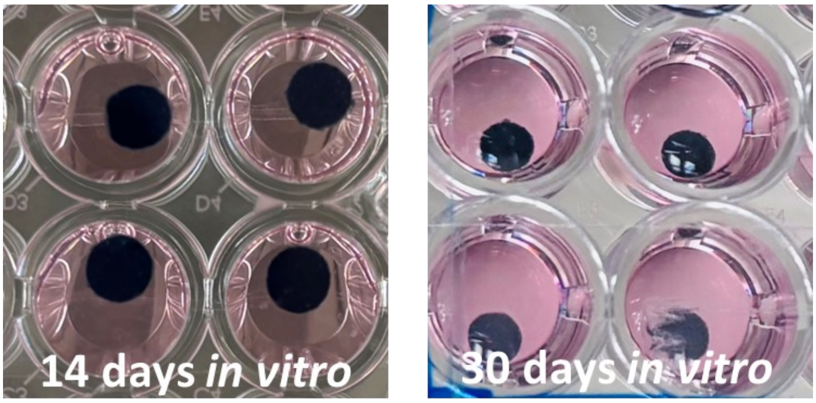

Figure S8: Electrically acive hydrogels in well plates incubated in cell media in a cell incubator for 14 days and 30 days

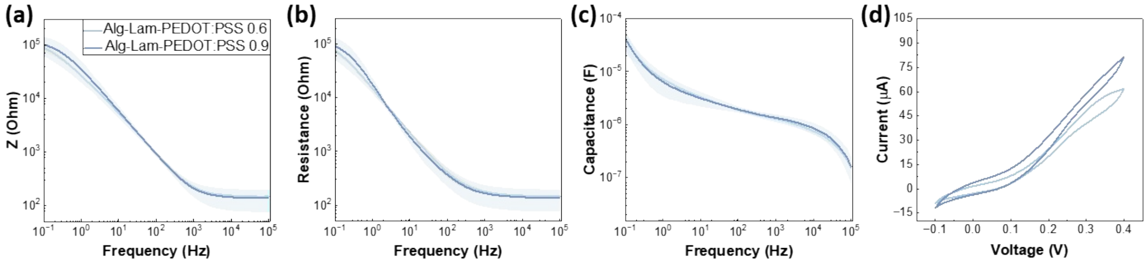

Figure S9: a) The impedance magnitude b) resitance c) capactiance verus frequency of alg-Lam, Alg-Lam-PEDOT:PSS 0.6 and Alg-Lam-PEDOT:PSS 0.9 hydrogels and d) the corresponding cyclic voltammogramms obtained with a scan rate at 0.1 V/s. All hydrogels were measured with the setup shown in figure S3 in cell media.

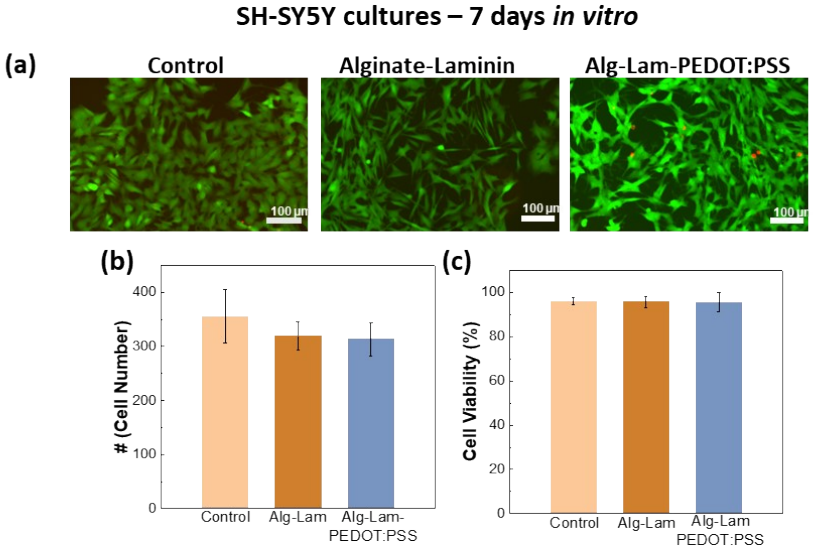

Figure S10: a) Live/Dead staining of SH-SY5Y neuroblastoma cells on Day 7 shows the impact on cell morphology and viability. Scale bar = 100 µm. From left to right - control cultures grown on polystyrene well plates, on polystyrene well plates together with Alg-Lam hydrogels and on polystyrene well plates together with Alg-Lam-PEDOT:PSS hydrogels. b) Average live cell count and (c) percentage of cell viability of SH-SY5Y neuroblastoma cells directly exposed to the alginate-laminin and alginate-laminin-PEDOT:PSS hydrogels.
